# Value of cytopathology in the diagnosis of adenoid cystic carcinoma and an analysis of misdiagnoses

**DOI:** 10.1186/s12893-023-01945-4

**Published:** 2023-03-09

**Authors:** Yu Wan, Changhai Long, Yun Liu, Jieqiong Wang, Xiaoqin Tang, Shaohua Wang

**Affiliations:** Department of Pathology, The Affiliated Hospital of Southwest Medical University, # No. 25, Taiping Street, 646000 Luzhou, Sichuan People’s Republic of China

**Keywords:** Adenoid cystic carcinoma, Cytopathology, Cytologic techniques, Histopathology, Retrospective studies

## Abstract

**Background:**

The diagnosis of adenoid cystic carcinoma (ACC) by cytopathology can be challenging. This study was aimed at testing the effectiveness of this technique and at assessing possible differences in the coincidence rate of fine-needle aspiration cytology(FNAC) and brush exfoliation.

**Methods:**

The pathology database of Southwest Medical University( Luzhou, China) was searched for patients who had undergone surgery or biopsy for ACC between January 2017 and January 2022 and had preoperative cytopathologic results. Their cytologic and histologic data were then analyzed retrospectively and the coincidence rates of cytopathology in the diagnosis of ACC were calculated.

**Results:**

Compared with histopathology, the total coincidence rate of the cytologic diagnosis of ACC was 76.8%, that of FNAC was 78.9%, and that of brush exfoliation was 55.6%.

**Conclusion:**

In the diagnosis of ACC, cytopathology is an effective tool; this is especially true of FNAC, which plays an important role in the diagnosis of ACC. The authors further suggest that it is advisable for diagnosticians to master the cytopathological features of ACC to reduce the possibility of preoperative misdiagnoses.

## Background

ACC accounts for 14.5% of all salivary gland tumors and 41% of malignant tumors of the large and small salivary glands [1, 2]. It can be found at various anatomic sites including the major and minor salivary glands, lacrimal glands, digestive tract, respiratory tract, skin, and breast. The diagnosis of ACC is challenging because this entity is composed of ductal epithelial cells and myoepithelial cells that manifest a variety of histologic characteristics [3]. This is, among other things, due to the overlap in cell types among different salivary gland tumors and the fact that additional staining is often of little help. Because of the technical limitations of cytopathology, it is also impossible to determine signs of malignancy, such as invasive growth and perineural or vasoinvasive growth [4]. Moreover, there is always the possibility of a sampling error.

FNAC is a convenient, rapid, and economical technique for the diagnosis of ACC. It is a simple procedure, most of which, depending on the location of the lesion, can be conducted by palpation or with imaging-guided assistance in the outpatient department; it also has a short turnaround time and is less invasive than biopsy [5, 6]. Once the aspiration has been done, the diagnosis can be determined quickly, thus allowing for timely and precise treatment [7]. FNAC is conventionally used to sample ACC from the salivary glands and other body surface masses, but it is rarely used in the airway and other deep masses. Bronchial lavage and brushing are commonly used cytologic examination methods for the airway and lung [8].

Preoperative biopsy of a malignant tumor can lead the tumor to proliferate; therefore cytopathologic examination has become one of the important diagnostic methods for the preoperative diagnosis of malignant tumors. In some cases, however, ACC is readily misdiagnosed because of its special location, atypical clinical symptoms, and possibly the physician’s inadequate understanding of the disease. In the present study, cytopathologic specimens of ACC confirmed by histology within the past 5 years were retrospectively analyzed; the cytologic diagnoses were then reviewed and the causes of misdiagnosis found, allowing us to determine the correct diagnosis in each case.

## Methods

### Patients

This is a retrospective study; thus, in all these cases, FNAC was done with the patients’ written consent. The departmental ethics committee also approved of the study. The pathology database of Southwest Medical University was consulted to search for patients who had undergone surgery or biopsy for ACC from January 2017 to January 2022 and had preoperative cytopathologic results. Information on age, date of examination, and side of the lesion was included in the database. We found 149 patients who had been treated for histopathologically confirmed ACC in this pathologic database, of whom 107 had received cytologic diagnostic results prior to surgery. The cytopathology smears of four cases lacked sufficient diagnostic material even on review and were therefore excluded from our study; four additional cases were excluded owing to imperfect cytologic slides that were no longer available for revision. Thus 99 cases with satisfactory cytologic smears and histologic confirmation were included. Moreover, all patients had completed preoperative CT examination. Most patients with ACC have specific CT findings, while a minority of patients with early ACC have no specific CT findings.

In all of these 99 cases, all cytology was performed with the written consent of the patient. The study was approved by the ethics committee of the Southwest Medical University (NO.:20220819-007), Luzhou, China. Because of the anonymous nature of the patient data and the study’s retrospective nature, it did not fall under the jurisdiction of the Medical Research Involving Human Subjects Act.

### Categorization and analysis

One of the investigators (YW) retrospectively classified the cytopathological reports. In case of uncertainty, a second reviewer (CH) was consulted. Each cytopathologic result was compared with the definitive histopathologic diagnosis as the gold standard; if the result was discordant or concordant with the cytopathologic result, this was noted. In case of revision of the histopathologic diagnosis, the revised diagnosis was considered the gold standard. If the two were judged to be inconsistent, a statistical analysis was conducted to analyze the causes of the misdiagnosis.

### Statistical analysis

We collected data using the Microsoft Excel application. All data are given as mean + standard deviation. We used SPSS software version 17.0 for Windows (IBM Corp, Armonk, New York) for statistical analyses. Diagnostic yields were compared using McNemar test. *P* < .05 was considered statistically significant.

## Results

### Clinical presentation and analysis of cytologic diagnosis

A total of 99 eligible cases were collected from the pathology database of Southwest Medical University, Luzhou, China, including 45 males and 54 females with an average age of 55.3 ± 11.6 years. The data of the study population are shown in Table 1. The coincidence rate of FNAC diagnosis of ACC was 78.9% and that of brush exfoliative cytology diagnosis of ACC was 55.6%, with a statistically significant difference (*P* < .01), as shown in Table 2. A total of 10 cases of ACC were misdiagnosed as other malignant tumors by cytology, of which 3 were misdiagnosed as lung adenocarcinoma, 2 as carcinoma ex pleomorphic adenoma, 3 as mucoepidermoid carcinoma, 1 as small cell lung cancer, and 1 as acinar cell carcinoma of the salivary gland. A total of 13 cases of ACC were misdiagnosed as benign lesions by cytology, of which 10 were misdiagnosed as pleomorphic adenoma and 3 as basal cell adenoma. Further analysis showed that, taking the final biopsy diagnosis as the reference, the coincidence rate of FNAC diagnosis as malignant was 90.0% and that of brush exfoliative cytology diagnosis as malignant was 88.9%, both of which were significantly improved (*P* < .01).

**Table 1:** Data obtained from 99 patients

Variables	*N* (%) or mean + SD
Age, years	55.3 ± 11.6
Gender	
Male	45 (45.5%)
Female	54 (54.5%)
Cytopathologic diagnosis	
ACC	76 (76.8%)
FNAC diagnosis: suspected ACC or ACC	71 (71.7%)
Brush exfoliative cytology diagnosis: suspected ACC or ACC	5 (5.1%)
Other malignancies	9 (9.1%)
FNAC diagnosis: other malignancies	6 (6.1%)
Brush exfoliative cytology diagnosis: other malignancies	3 (3.0%)
Benign disease	14 (14.1%)
FNAC diagnosis: benign disease	13 (13.1%)
Brush exfoliative cytology diagnosis: benign disease	1 (1.0%)

ACC, adenoid cystic carcinoma; FNAC, fine-needle aspiration cytology; SD, standard deviation

**Table 2:** Diagnostic yields of cytopathologic diagnosis

Cytopathologic diagnosis	*n*	FNAC (*n*, %)	Brush exfoliative cytology (*n*, %)
suspected ACC or actual ACC	76	71 (78.9)	5 (55.6)*
MEC	3	3 (3.3)	0
LUAD	2	0	2 (22.2)
Ca-ex-PA	2	2(2.2)	0
SCLC	1	0	1 (11.1)
AciCC	1	1 (1.1)	0
PA	10	10 (11.1)	0
BCA	3	3 (3.3)	0
Chronic inflammatory reaction	1	0	1 (11.1)
Total	99	90	9

ACC, adenoid cystic carcinoma; AciCC, acinar cell carcinoma of the salivary gland; BCA, basal cell adenoma; Ca-ex-Pa, carcinoma ex pleomorphic adenoma; LUAD, lung adenocarcinoma; MEC, mucoepidermoid carcinoma; PA, pleomorphic adenoma; SCLC, small-cell lung cancer

*P < .01 vs FNAC

### Cytomorphologic findings

**Fig. 1:**
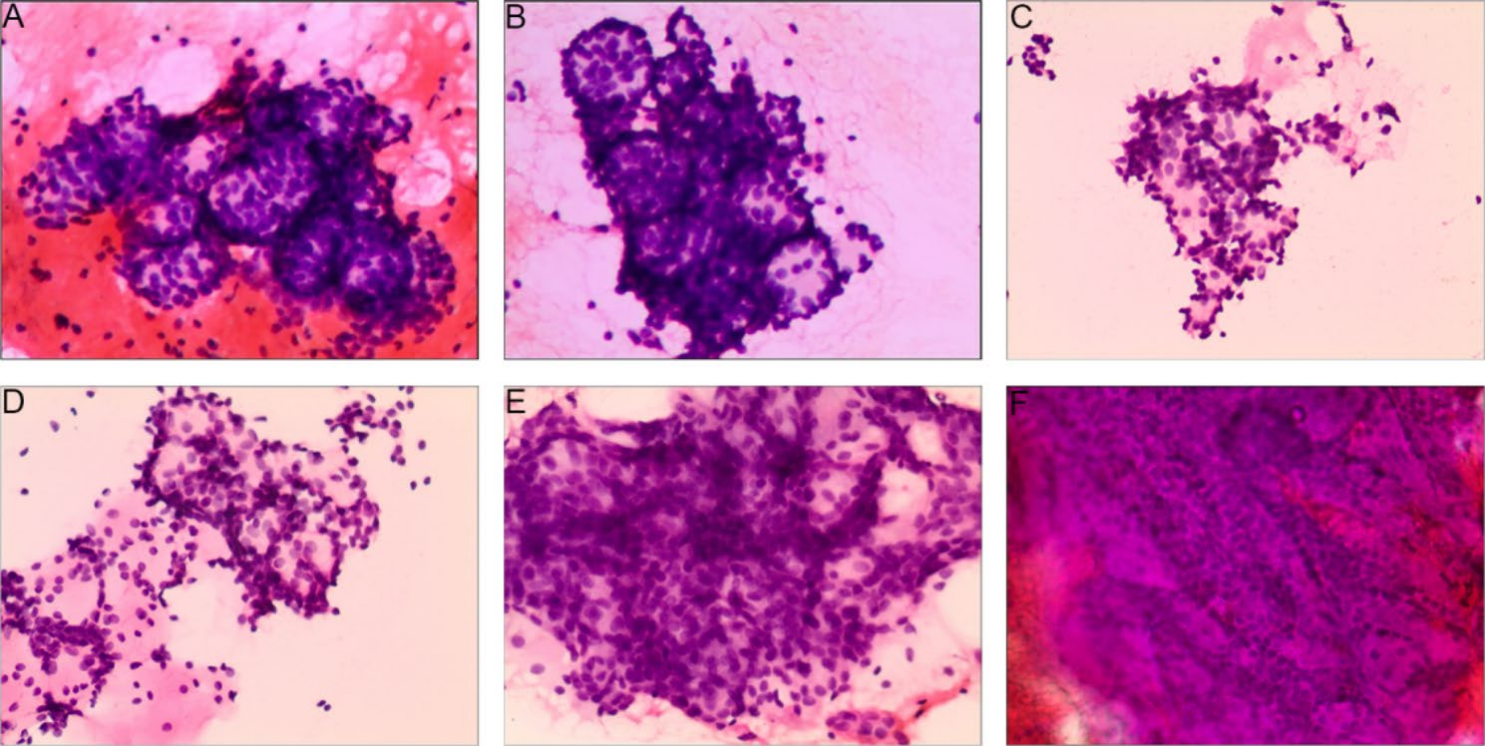
Cytomorphologic characteristics of adenoid cystic carcinoma (ACC). (A and B) Cribriform vesicle structures of different sizes (H&E stain, ×100). (C, D) Translucent spherical body surrounded by tumor cells (H&E stain, ×100). (E) Moderately atypical basal-like cells arranged in a three-dimensional pattern (H&E stain, ×100). (F) Cells arranged in stripes, like the layers of waves hitting a beach (H&E stain, ×100)

The most prominent cytologic features of ACC are a cribriform vesicular structure (Fig. 1A and B) and a translucent spherical body surrounded by tumor cells (Fig. 1C and D). This type of mucous ball is found only in ACC and not in other types of salivary gland tumors; thus it has great diagnostic value. The cytomorphologic characteristics of our 99 cases were reviewed again, as shown in Table 3. In the cytologic smear of ACC, the number of cells is large and the volume is small. The size and morphology of cancer cells are basically the same. The nuclei are mostly round and oval, generally without significant atypia. The tumor cells are often densely distributed in irregular masses or scattered, while the nuclear chromatin is rough and dense, with small nucleoli. There is little basophilic cytoplasm. Sometimes the basal-like cells are seen to be moderately atypical and arranged in three dimensions (Fig. 1E). Cells arranged in stripes may also be seen, resembling the layered structure of waves hitting a beach (Fig. 1F).

**Table 3:** Spectrum of cytologic features of 99 cases of adenoid cystic carcinoma

Cytopathologic features	*n*	Tumor cells	Total
Cell amount	71	Many, > 50%	71/99
	23	Moderate, 25–50%	23/99
	5	Few, < 25%	5/99
Chromatin	95	Coarse	95/99
	4	Fine	4/99
Nucleoli	93	Absent	93/99
	6	Present	6/99
Cytoplasm	96	Scant	96/99
	3	Moderate	3/99
Hyaline globules	96	Present	96/99
	3	Absent	3/99

### Histopathologic findings

**Fig. 2:**
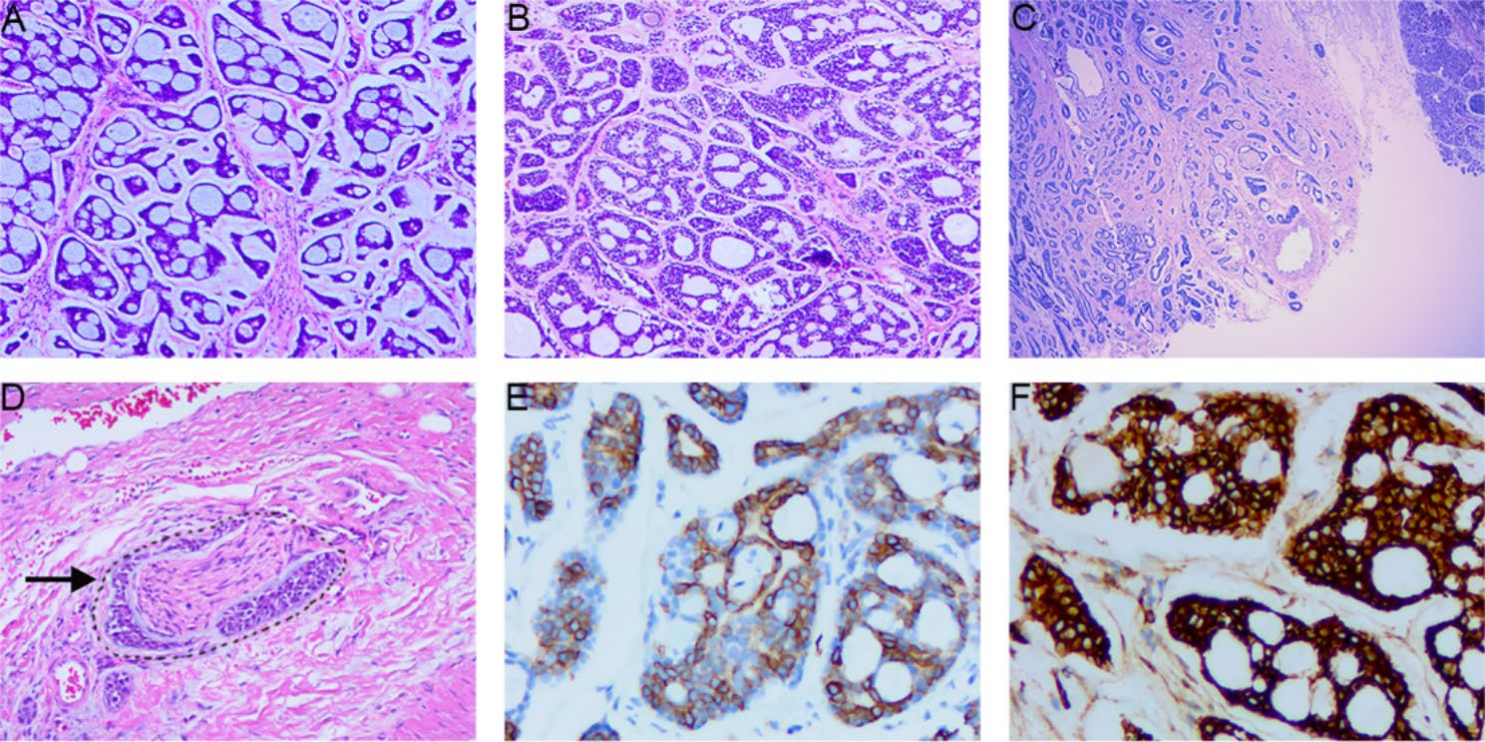
Histopathologic characteristics of adenoid cystic carcinoma (ACC). (A and B) Typical sieve-like structure (H&E stain, ×100). (C) The glands are arranged in a tubular manner (H&E stain, ×40). (D) Invasion of nerve by cancer cells (H&E stain, ×200). (E) Immunohistochemical staining [CD117(+)]. (F) Immunohistochemical staining [BCL-2(+)].

An ACC is composed of glandular epithelial cells and myoepithelial cells, both of which are small; they have hyperchromatic nuclei, no obvious nuclear atypia, rare mitotic images, and little cytoplasm. The glandular cells are arranged in tubes, and myoepithelial cells account for the majority of the tumor. These cells are often flaky or located at the periphery of the tubular structure; their nuclei are often angular. There are three types of tumor arrangements: cribriform (Fig. 2A and B), tubular (Fig. 2C) and solid. Sometimes all three can be seen within the same tumor. ACC often invades nerves (Fig. 2D). Immunohistochemistry showed that CD117 (Fig. 2E), BCL-2 (Fig. 2F), P16, and Ki-67 were positive.

## Discussion

ACC is a slow-growing tumor that commonly remains asymptomatic for a long time before it is diagnosed [9, 10]. Cytopathologic examination is the standard diagnostic test in the management of ACC, but, as illustrated previously, this technique is known to have its limitations. Fortunately, cytopathologists are gradually learning more about the cytologic variations of ACC and thus are making fewer diagnostic mistakes.

ACC in its early stage can invade the nerve bundle, but its expansion is slow [11–13]. It often invades the surrounding tissues and, in the late stage, metastasizes to the locally draining lymph nodes, lungs, or bones. Early diagnosis has a significant impact on the treatment and prognosis of this tumor, and FNAC is superior to biopsy in this respect. FNAC has the advantages of causing little damage and having few complications, good repeatability, and no risk of needle transfer [14]. FNAC and brush exfoliative cytology examination of all ACCs in this study led to no serious complications. Interestingly, although all patients in this study completed CT or PET/CT examination before surgery, no correlation between cytopathological findings and CT examination was found.The reason may be related to the small number of patients enrolled in this study. Future work in this field requires more center studies to clarify the relationship between cytopathologic findings of ACC and CT examination.

ACC, like other types of malignant tumors, is difficult to diagnose preoperatively, but cytologic examination can be done for further diagnosis [15, 16]. Among the 99 patients included in this study, 76 were diagnosed with ACC preoperatively, with an accuracy rate of 76.8%. However, the coincidence rate of cytologic diagnosis based on brush exfoliation was 55.6%. This is obviously lower than that found in the study of salivary gland FNAC. We have found that the cells of ACC obtained by brush cytology were small under the microscope, their nuclear chromatin was fine and granular; moreover, heteromorphism was not obvious and the cells sometimes lacked the typical morphologic characteristics. Especially in the trachea and bronchi, ACC is usually covered by intact mucous membranes, and no tumor cells are found in bronchial cytological specimens. In rare cases, tumor infiltrates the overlying mucosa, and tumor cells can be seen in bronchial rinse or bronchial brush specimens. These features make Brush cytology for ACC easy to be misdiagnosed or missed. Further analysis showed that this difference was related to specimen fixation. Brush cytology is completed under fiberbronchoscope, which is difficult to operate and takes a long time, so it is difficult to fix the sample in time, and cell degeneration will occur. In cases that raise suspicion of ACC, additional immunohistochemical (BCL-2, P63, CD117) or FISH (MYB) can be applied to cell blocks to refine the diagnosis. Further analysis showed that the diagnostic rate of preoperative cytologic examination in differentiating benign and malignant tumors was about 85.9%. Compared with the rapidly frozen pathologic section obtained during surgery, cytologic examination has not only high diagnostic accuracy but also an important advantage in preoperative diagnosis [17]. In particular, the accuracy of FNAC in identifying malignant tumors is close to that of intraoperative frozen Sect. [18]. It is a simple procedure, most of which, depending on the location of the lesion, can be conducted by palpation or with imaging-guided assistance in the outpatient department; it also has a short turnaround time and is less invasive than biopsy. The diagnosis can be attained quickly after the aspiration is done, thus allowing instant planning of the patient’s care.

In the course of our study, we found that cytologic examination has also missed diagnoses or led to misdiagnosis. We carefully analyzed the causes and found that the following problems may arise: (1)When the tumor is small and the cytologic sampling technology poor, the most diagnostically significant tumor components may not be observed. (2) Pleomorphic adenoma (PA) and basal cell adenoma are common benign tumors of the salivary gland that are similar to ACC in cell morphology, Therefore ACC can appear in the same mass as pleomorphic adenoma and have similar morphologic manifestations in cytologic smears. (3)The morphologic differentiation of tumor cells may be good but the heterotype is small, so that it is difficult to distinguish from a benign tumor. (4)Careless reading of the film, insufficient diagnostic experience, and the mistaken identification of diagnostically important cells can lead to error. However, on review and after a careful search, small remnants of mucoid stromal balls may be seen. In this case, the misdiagnosis would have been due to the lack of care and failure to understand this feature.

Discriminating between ACC and PA sometimes poses the greatest difficulty [19–21]. Both of these tumors may present as pseudocolumnar tumors and pseudotrabecular structures, leading to misdiagnosis. In addition, artificial features—such as the transformation of the transparent sphere of ACC into a rectangular structure (due to the traumatic effect of suction and sliding operations)—can simulate the trabecular aspect of the mucilaginous matrix component of PA and thus may also lead to a false diagnosis. In this study, 10 cases of ACC were initially misdiagnosed as PA.

## Conclusion

The preoperative diagnosis of ACC has important clinical significance for prognosis and treatment. Our study once again proved the validity and effectiveness of cytopathology in the diagnosis of ACC. Therefore, we strongly recommend the use of this diagnostic tool to diagnose ACC before operation. However, the number of patients participating in this study is not conspicuous, and we will reserve future insights on larger samples also through collaborations with Centers where greater numbers. It is difficult to arrive at a preoperative diagnosis of ACC because its most prominent cytologic feature (i.e., the translucent spherical body whose surface is surrounded by tumor cells) is not always reported to be present on cytology specimens. We believe that diagnosis of ACC improves with the awareness and experience of the cytopathologist.

## Data Availability

The datasets generated and/or analyzed during the current study are not publicly available as they consist of confdential patient data; however, data will be made available from the corresponding author on reasonable request.

## Abbreviations

ACC: Adenoid cystic carcinoma
FNAC: Fine-needle aspiration cytology
AciCC: Acinar cell carcinoma of the salivary gland
BCA: Basal cell adenoma
Ca-ex-Pa: Carcinoma ex pleomorphic adenoma
LUAD: Lung adenocarcinoma
MEC: Mucoepidermoid carcinoma
PA: Pleomorphic adenoma
SCLC: Small-cell lung cancer
